# HerediVar and HerediClassify: tools for streamlining genetic variant classification in hereditary breast and ovarian cancer

**DOI:** 10.1186/s40246-025-00787-w

**Published:** 2025-07-04

**Authors:** Anna-Lena Katzke, Marvin Doebel, Jan Hauke, Gunnar Schmidt, Marc Sturm

**Affiliations:** 1 Department of Human Genetics, Hannover Medical School, Hannover, Germany; 2 Institute of Medical Genetics and Applied Genomics, University of Tübingen, Tübingen, Germany; 3 Medical Faculty, Center for Hereditary Breast and Ovarian Cancer, Center for Integrated Oncology (CIO), University Hospital Cologne, Cologne, Germany

**Keywords:** Automated variant classification, Variant classification, Variant annotation, Hereditary breast and ovarian cancer, ACMG, Genetic diagnostics

## Abstract

**Background:**

Multiple different evidence types as well as gene-specific variant classification guidelines need to be considered during the classification of variants, making the process complex. Therefore, tools that support variant classification by experts are urgently needed.

**Methods:**

We present HerediVar a web application and HerediClassify a variant classification algorithm. The performance of HerediClassify was validated and compared to other variant classification tools. HerediClassify implements 19/28 variant classification criteria by the American College of Medical Genetics and gene-specific recommendations for *ATM*, *BRCA1*, *BRCA2*, *CDH1*, *PALB2*, *PTEN*, and *TP53*.

**Results:**

HerediVar offers modular annotation services and allows for collaboration in the classification of variants. On the validation dataset, HerediClassify shows an average F1-Score of 93% across all criteria. HerediClassify outperforms other automated variant classification tools like vaRHC and Cancer SIGVAR.

**Conclusion:**

In HerediVar and HerediClassify we present a powerful solution to support variant classification in HBOC. Through their modular design, HerediVar and HerediClassify are easily extendable to other use cases and human genetic diagnostics as a whole.

## Introduction

According to the World Health Organization (WHO) breast cancer is the most common cancer type among women with over 2 million new cases diagnosed in 2022 worldwide [41]. Approximately 10–15% of all breast cancer cases are associated with hereditary breast and ovarian cancer (HBOC) [14, 23]. Ovarian cancer is less common than breast cancer with 300,000 cases diagnosed globally in 2022 [20], around 20% of cases are estimated to be caused by genetic variants in HBOC related genes [28].

Genetic testing for patients with a familial predisposition to breast cancer has become more accessible and affordable due to next generation sequencing (NGS), causing the bottleneck in genetic diagnostics to shift from sequencing to variant interpretation. The classification of variants is a time-consuming process due to the diverse and complex types of evidence, including population data, allelic evidence, cosegregation data, computational prediction algorithms, and medical literature [33]. The American College of Medical Genetics and Genomics and the Association of Molecular Pathologists (ACMG/AMP) guidelines for variant classification define 28 criteria to assess these types of evidence [33]. In addition to the ACMG/AMP guidelines, gene-specific recommendations as published for *BRCA1* and *BRCA2* [30], *ATM* [13], and *TP53* [15] further increase the complexity of variant classification.

Due to changes in recommendations and updates to databases and prediction tools, variants should be reclassified at regular intervals [40]. Reclassification is especially important for variants of uncertain significance (VUS) which are defined as variants whose impact on disease is unknown. Due to the large number of variants to reclassify, with 10–20% of variants classified as VUS in *BRCA1* and *BRCA2* alone, manual reclassification is not possible [12]. The point-based system, proposed by Tavtigian et al. [37], enables the identification and prioritization of VUS, by highlighting variants with a score close to the likely benign or likely pathogenic cut-off. Focusing on these variants for reclassification presents an opportunity to increase the efficiency of variant reclassification.

We and others found that classification varies among clinical experts, albeit using the same set of criteria [3]. Reasons for discrepancies in variant classification using the ACMG/AMP guidelines are that variant classification relies on many different evidence types, which are scattered across databases or publications. This presents an enormous effort to find relevant information. Further discrepancies arise due to varying interpreter opinions or differences in the application of criteria. The latter is further enhanced by the fact that the strength of individual criteria may be adjusted based on the strength of evidence. Nevertheless, these issues can be alleviated through structured collaboration between groups.

To address these needs, clinical experts require highly modular and accessible software tools. Existing tools like CanVarUK [6] or GoldenHelix (Golden Helix, Inc.) are restricted by a paywall, and lack essential features such as modular variant annotation and the implementation of gene-specific classification schemes. Further, they do not allow for consensus classifications among experts.

Several automated variant classification tools have been published, including vaRHC [27] and Cancer SIGVAR [24], which incorporate gene-specific recommendations for cancer risk genes. These tools do not implement the *BRCA1* and *BRCA2* guidelines, which are essential for variant classification in HBOC.

In order to support experts in variant classification and reclassification, we developed the web application, HerediVar, and an automated variant classification algorithm, HerediClassify. These projects were a key part of the HerediVar project funded by German Cancer Aid. Through this project, clinical data and functional analysis will become available to support variant classification. HerediVar enables the collaborative classification of genetic variants by automating variant curation, annotation and classification through HerediClassify as well as highlighting variants through rich filtering and sorting options. HerediClassify implements gene-specific guidelines for HBOC risk genes and is designed for easy extensibility to accommodate future updates or novel gene-specific variant classification guidelines.

The live version of HerediVar is available online at https://heredivar.uni-koeln.de. To date, HerediVar is actively used by clinical scientists across 26 genomic research centers in Germany and continues to grow. The source code can be found on GitHub (https://github.com/GC-HBOC/HerediVar and https://github.com/akatzke/HerediClassify).

## Methods

### Web application architecture and deployment infrastructure

The HerediVar backend uses the python micro web framework flask. Authentication is done through the state-of-the-art OAuth2 provider, Keycloak. Long-running tasks are delegated to a background job-processing queue implemented through Celery alongside a Redis database. The HerediVar frontend mainly uses JavaScript with jQuery. More information about the web application architecture and installation instructions can be found on the HerediVar GitHub page (https://github.com/GC-HBOC/HerediVar).

### Variant curation

When importing variants into HerediVar the chromosome, reference, and alternative sequence are checked for validity. Furthermore, variant comparability is ensured by left alignment. These steps are performed with the VcfCheck and VcfLeftNormalize tools of the ngs-bits toolbox [36]. Lifting variants from GRCh37 to the GRCh38 reference genome is performed with CrossMap (v.0.6.5) along with the Ensembl GRCh37 to GRCh38 chainfile. Often variants are not available in variant call format (VCF) style, but rather as Human Genome Variation Society (HGVS).c strings. In these cases, HerediVar recovers the VCF style using the HgvsToVcf tool from ngs-bits.

### HerediClassify

HerediClassify is an open-source Python-based variant classification algorithm implementing the ACMG/AMP classification criteria [33]. Where possible, code from the GenOtoScope tool for automated variant classification in hearing loss has been integrated into HerediClassify, undergoing refactoring if necessary [26].

### Classification guidelines

HerediVar and HerediClassify support the current ACMG/AMP classification guidelines as well as gene-specific guidelines for *ATM* (v.1.3.0), *BRCA1* (v.1.1.0), *BRCA2* (v.1.1.0), *CDH1* (v.3.1.0), *PALB2* (v.1.1.0), *PTEN* (v.3.1.0), and *TP53* (v.1.4.0) (ClinGen [9] accessed May 2024).

**Fig. 1:**
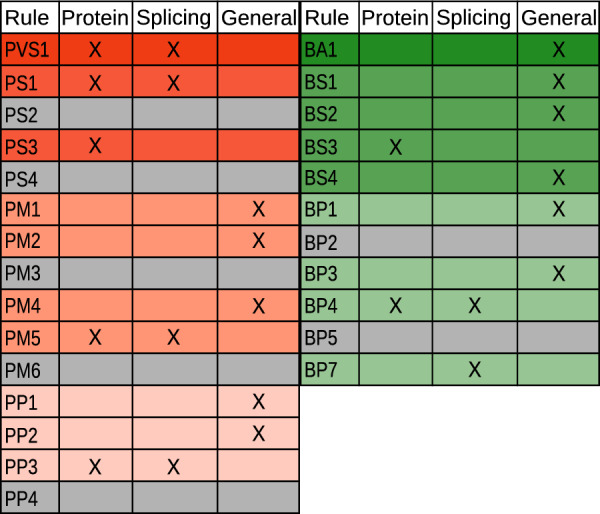
Overview of all rules, excluding BP6 and PP5, and their possible evidence types. Rules in gray are not implemented in HerediClassify

HerediClassify implements automatic selection of 19 out of the 28 ACMG/AMP criteria (Fig.1). The criteria assessing de novo data (PS2, PM6), allelic data (PM3, BP2, BP5), phenotype (PP4), and enrichment of variants in a disease population (PS4) are not implemented as machine-readable data is not available. The ClinGen recommendations for the application of PVS1 [1], PM2, PP3/BP4 [32], and PP5/BP6 [4] have been implemented. As proposed by Walker et al. [39], splicing specific updates to PVS1, PS1, PP3, BP4, and BP7 were implemented. Therefore, HerediClassify generates two classifications per variant: one for protein-level and one for splicing-level variant effect. This is achieved by assigning every rule an evidence type: “general”, “protein” or “splicing” (Fig.1). For the classification of the variant effect on splicing, criteria with the evidence type “general” and “splicing” are combined. Likewise, “general” and “protein” evidence are combined for the classification on protein level.

In accordance with recommendations for PP3/BP4, only one prediction tool is used for computational evidence [32]. For splicing prediction, SpliceAI is used for all genes and REVEL is used for pathogenicity prediction in all genes except *BRCA1*, *BRCA2*, and *TP53* where guidelines recommend BayesDel.

For the final classification of the variant, both the original schema proposed in the ACMG guidelines[33] and the point-based system as proposed by Tavtigian et al. [37] are implemented in HerediVar and HerediClassify.

### Modularity

HerediVar and HerediClassify were designed for modularity and customizability. Configuration files allow users to adjust annotations in HerediVar or set thresholds for computational evidence criteria in HerediClassify. Uniquely, HerediClassify allows the user to select the rules to be applied from a list of implemented rules.

### Validation and benchmarking datasets

For the validation of HerediClassify 727 variants were selected from the ClinGen Evidence Repository (accessed February 2024). 6 variants were excluded, as variants larger than 15 bp fall outside the scope of HerediClassify. The final validation dataset consists of 721 variants in *ATM* (n = 31), *BRCA1* (n = 18), *BRCA2* (n = 20), *CDH1* (n = 321), *PALB2* (n = 38), *PTEN* (n = 175), and *TP53* (n = 118). Reannotation with gnomAD v2.1.1. was performed for the validation dataset.

Between October 2023 and May 2024, a benchmarking dataset consisting of 142 variants classified by the VUS task force, an expert committee for variant classification of the German Consortium for Hereditary Breast and Ovarian Cancer (GC-HBOC) was collected. 12 variants that were in the ClinGen Evidence Repository data set were excluded from the HerediVar dataset, bringing the benchmarking dataset to 130 variants (Supplemental Figure 1). This dataset was used for comparing HerediClassify to the following tools: vaRHC [27], TAPES [42], InterVar [25], VarSome [22], and Cancer SIGVAR [24].

All tools were validated rule by rule, considering the applied evidence strengths. For rules that assess both splicing and protein evidence, results were summarized for comparison to the ground truth. For pathogenic evidence criteria, triggering of either the protein or splice implementation is sufficient to count a rule as called. In case of benign evidence criteria, both protein and splicing implementation of the rule need to be applied in order for the rule to be called. For both datasets, recall and precision were calculated using scikit (v.1.4.2).

For the comparison of the consensus classifications in HerediVar to the classification by HerediClassify, the protein and splicing evidence-based classification were summarized into one final classification. When one classification was VUS and the other not, the non-VUS classification was the final classification. In case a benign and a pathogenic classification were present, the final classification of the variant was VUS.

### Annotation service architecture

The HerediVar annotation service is a versatile tool for variant annotation, which allows rapid adaption to the fast development of variant annotation sources. The annotation service picks up annotation job objects and executes them sequentially for every variant. Currently, the annotation service is capable of annotating various pathogenicity predictors, splicing scores, external classifications, population scores, protein domain sources, literature annotation tools, and more. All databases are downloaded and preprocessed using the same pipeline as for variant import to ensure matching variants between HerediVar and external sources (Supplemental Table 1).

### HerediCaRe database & API

The The Hereditary Cancer Registry (HerediCaRe) database is used by affiliated centers of the GC-HBOC for long-term documentation of clinical and genetic data [11]. HerediCaRe collects data from families with a history of breast and ovarian cancer, recording clinical data, genetic diagnostics, and the presence of cancer risk factors within each family. Trusted centers can upload patient-related variants and assessments of pathogenicity. After authentication, this information can be downloaded through a novel Application Programming Interface (API). The API allows posting new variants and classifications.

HerediCaRe is hosted at the Institute of Medical Informatics, Statistics and Epidemiology at the University of Leipzig since 2019 and was established in cooperation with certified cancer centers, the statutory health insurance companies, the German Cancer Society (DKG) and the self-help group BRCA Network.

## Results

### Overview

HerediVar is a web application for automatic and manually curated classification of variants that are associated with HBOC. It is designed to be a one-stop platform for variant annotation, interpretation, and classification, which is stable and easy to maintain. Clinical scientists can collaborate in variant classification and create consensus classifications. Although the database has public read access, only members of the GC-HBOC are allowed to submit data, to ensure high data quality.

HerediVar communicates closely with HerediCaRe, a clinical database hosted in Leipzig for variant import. Classifications can be uploaded to HerediCaRe via an API. HerediCaRe maps variants to patients, enabling swift recontacting of patients in case a variant is re-evaluated. HerediVar variant classification data can also be downloaded in VCF format.

A diverse set of utility features are available, like gathering sets of variants in user-defined lists, uploading functional assays, publishing classifications to ClinVar and automated variant classification. A wide range of administrative options are supported, including data statistics, status of variant annotations, and bulk re-annotation. HerediVar is built in a highly modular way, such that, new annotations or classification schemes can be added easily. A rich documentation can be found on the HerediVar web interface, which guides the user through all features.

The production installation of HerediVar is hosted at the University of Cologne (https://heredivar.uni-koeln.de/). It consists of a backend server that processes and stores the data and serves the client webpages. Long-running tasks, such as variant annotations, are delegated to a background task queue. HerediVar provides a highly secure user role setup with associated authentication and authorization. It also protects against common attack vectors such as cross-site scripting and injection attacks.

### Workflow and interface

The typical workflow starts with the import of variants from HerediCaRe or by manually adding variants through the web interface. Small variants can be added by genomic location with reference and alternative alleles, or by cDNA position in HGVS format with gene or transcript specification.

HerediVar always checks the input, making sure that only correct variants are inserted. Subsequently, all variants are mapped to the reference genome GRCh38 and normalized to ensure consistent variant representation and comparability.

Each new variant is automatically annotated with meta information. The annotation is processed in the background to ensure that the frontend stays responsive. This service annotates the variant with a wide variety of data, including *in-silico* pathogenicity prediction scores, splicing prediction scores, third-party classifications, population scores, protein domains and others. These include CADD, REVEL, SpliceAI, Hexplorer, ClinVar, gnomAD, PFAM, Cancerhotspots, and VEP (Supplemental Table 1).

A main feature of HerediVar is the integration of the automatic variant classification algorithm HerediClassify that follows the ACMG/AMP guidelines [33]. HerediClassify starts after all annotations are processed and computes a preselection of ACMG/AMP criteria.

Once variants are inserted, the user can navigate to the browse-variants-page. This shows a multi-page table of all available variants currently known by HerediVar. This table provides a first impression of all variants by showing positional information along with classifications by GC-HBOC experts. Additionally, it offers rich filter options, for instance by classification result, gene, or genomic position as well as sorting options. To facilitate highlighting variants which are close to being reclassified from VUS to likely pathogenic or likely benign, this table can be filtered by the point-based scoring system [37]. Filters can also be saved and shared by copying the URL. Furthermore, users can create custom lists, which can be made accessible to others or kept private, facilitating the sharing and browsing of variants.

**Fig. 2:**
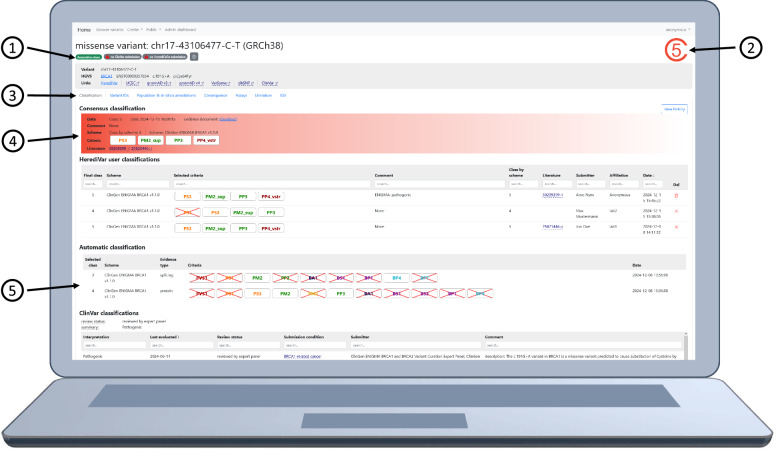
An exemplary view of the variant details page of rs55851803 in the HerediVar webapp. The depicted classification serves as an example and should not be used in clinical decision-making. This page consists of 1: The status bar. It shows errors and the upload status. This consensus classification was not uploaded to ClinVar or HerediCaRe yet. Among others, the gear-button provides links to reach the classify page and to upload the variant classification. 2: The consensus classification label. This variant was classified as pathogenic. 3: The navigation tabs. The classification tab is selected. They can be used to access further information about the variant e.g. SpliceAI scores in the Population & in-silico annotations tab. 4: The consensus classification details. 5: The HerediClassify results

Each row in the variant table can be clicked, which leads to the variant-details-page (Fig. 2). This page shows all available information about the variant. Annotations are structured in seven tabs: classification, variant IDs, population & *in-silico* annotations, consequence, assays, literature, and Integrative Genomics Viewer (IGV). The final tab has a customized igv.js browser that shows the location of the variant, other classified variants, and Ensembl transcripts. The variant details page provides reannotation of the variant, submission of splicing or functional assays, or export of the variant and its annotations to VCF.

In order to submit a new classification, the user has to navigate to the classify-variant-page (Supplemental Figure 2). HerediVar supports multiple different ACMG/AMP-based classification schemes. The webpage presents a clearly arranged set of color-coded criteria buttons. Each one can be clicked, which displays an explanation and advice on when to select the criterion. Importantly, HerediVar does not allow selection of criteria without providing evidence for them. Criteria can be marked as “not selected” if there is clear evidence against a criterion. A final class that might differ from the one automatically selected by the scheme along with a comment and literature can be provided. To facilitate criteria selection, the classify page allows preselecting criteria using HerediClassify. User classifications can be edited and deleted at any time. Consensus classifications cannot be deleted, but updated. HerediVar tracks the history of all previous consensus classifications. For later reference, each consensus classification comes with an evidence document reporting the annotation status at the time when the classification was submitted. Finally, consensus classifications can be published to ClinVar through an easy-to-use submission form directly from HerediVar. Public download is available for all consensus classifications on the HerediVar website. It is also possible to upload variants and their consensus classification to HerediCaRe.

### Implementation of HerediClassify

HerediClassify classifies variants up to 15 bp. It requires pre-annotated input, which HerediClassify receives through a REST-based API from HerediVar. The API can be easily adapted to accommodate VCF inputs and enables easy integration of HerediClassify into existing annotation workflows.

HerediClassify implements the variant classification schemes as defined in the ACMG/AMP guidelines and refined in the gene-specific guidelines. HerediClassify returns two final classifications for each variant, one based on protein evidence and one based on splicing evidence. Additionally, the assessment status of every rule is returned with a comment explaining why a rule has been applied.

### Validation of HerediClassify

**Fig. 3:**
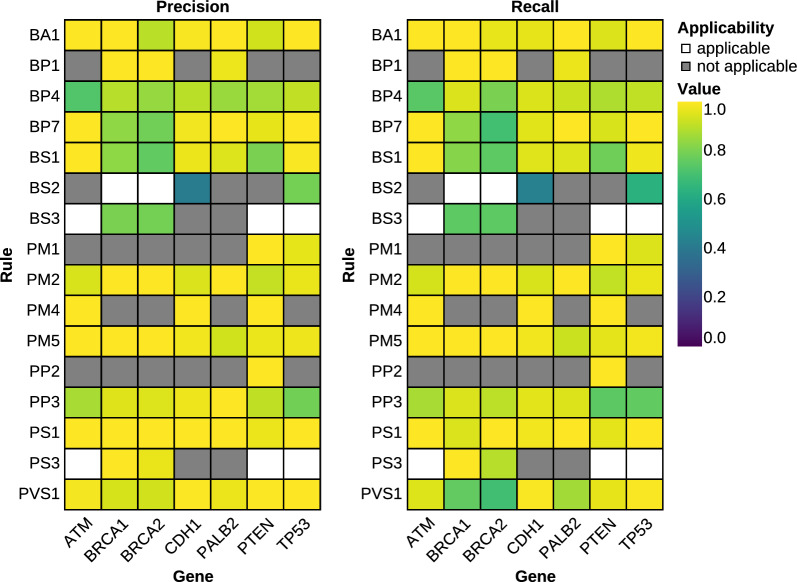
Heatmap showing precision (left) and recall (right) of HerediClassify on the validation dataset from the ClinGen Evidence Repository. The performance is depicted for every rule on a gene by gene level. Rules that are not applicable to the gene are colored in gray, rules that do apply but have not been implemented are shown in white. BP3 is not applicable according to all gene-specific guidelines. Results for PP1 and BS4 are excluded due to unavailability of data

HerediClassify was validated using 721 variants from the ClinGen Evidence Repository. Overall, HerediClassify shows good performance, with an average F1-score across all rules of 93% (Fig. 3).

For the annotation of variant population frequency, HerediVar utilizes gnomAD v3.1.2. For the classifications in the ClinGen Evidence Repository, gnomAD v2 was used. Therefore, we performed manual reannotation for the validation, with gnomAD v2.1.1 for the validation dataset. The application of criteria based on population frequency - BA1, BS1, and PM2 - differs between the ClinGen Evidence Repository and HerediClassify in 14% of cases (Supplemental Table 2).

PM2 was downgraded from moderate to supporting evidence strength in newer variant classification guidelines. Classifications in the ClinGen Evidence Repository, using the old evidence strength, were updated in line with current recommendations. This leads to a minimum F1-score of 92% in BA1, 78% in BS1 and 91% in PM2.

HerediClassify uses the FLOSSIES database for calling BS2. Therefore, BS2 is not implemented for *BRCA1*, *BRCA2*, *PALB2*, and *PTEN* where the gene-specific recommendations do not allow for the utilization of FLOSSIES. BS2 has a F1-score of 42% yin *CDH1* and 72% in *TP53*.

BS3 and PS3 are only implemented for *BRCA1* and *BRCA2*. PS3 has a minimum F1-score of 93%, and BS3 has a minimum F1-score of 77%.

The performance of PVS1 and BP7 is lower in *BRCA1* and *BRCA2* than in other genes, with a minimum F1-score of 80% for PVS1 and 74% for BP7. This is caused by the integration of RNA data into the assessment of PVS1 and BP7 for *BRCA1* and *BRCA2*. In all other genes, the F1-score is higher, with a minimum of 95% for BP7 and 91% for PVS1.

The assessment of computation evidence criteria is performed using one pathogenicity prediction tool and one splicing prediction tool. As gene-specific recommendations for *ATM*, *CDH1*, and *PTEN* call for multiple prediction tools, HerediClassify’s performance is affected, resulting in a minimum F1-score of 77% for PP3 and 74% for BP4. BP4 is considered applicable only if both splicing and pathogenicity prediction indicate benignity. In all but one case, where BP4 is applicable according to the ClinGen Evidence Repository, either the protein prediction or the splicing prediction is benign according to HerediClassify (Supplemental Table 3). In the one exception where HerediClassify does not call BP4, a splicing prediction by HumanSpliceFinder was used. Variants where splicing and pathogenicity prediction are contradictory, for instance often the case in synonymous variants, or one prediction is missing, for instance for intronic variants, BP4 performs particularly bad (Supplemental Figure 3). Excluding synonymous and missense variants, BP4 has a minimum F1-score of 95% across all variant types.

### Benchmarking of HerediClassify

**Fig. 4:**
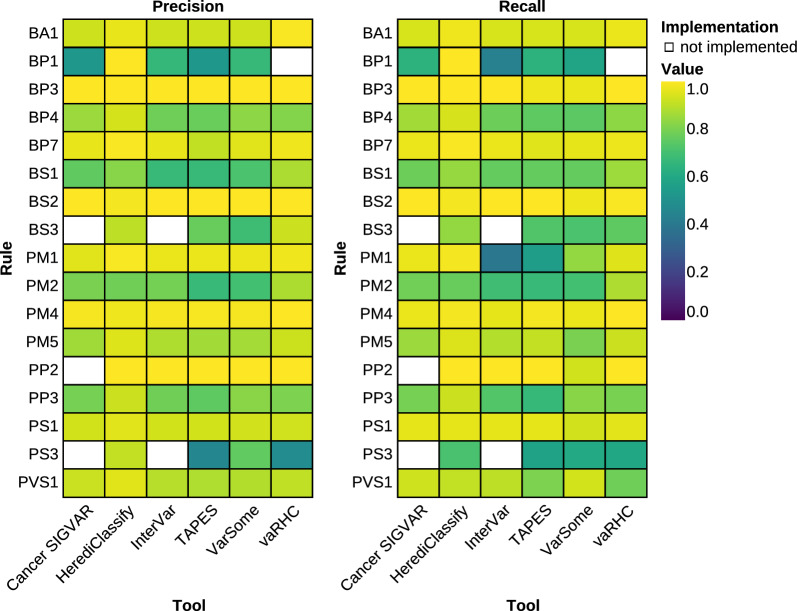
Heatmap showing precision (left) and recall (right) for HerediClassify, vaRHC, VarSome, Cancer SIGVAR, TAPES and InterVar for every rule. Performance metrics were calculated taking the evidence strength into account. White fields indicate rules that have not been implemented by the respective tool

To assess the performance of HerediClassify on a dataset that represents the variant distribution in a HBOC setting, variants classified by the VUS task force were collected. Based on this dataset comprised of 130 variants, the performance of HerediClassify was compared to other automated variant classification tools, including VarSome [22], TAPES [42], vaRHC [27], InterVar [25], and Cancer SIGVAR [24]. HerediClassify achieves a F1-score of 68% for variant classification, outperforming VarSome (63%), TAPES (51%), vaRHC (59%), InterVar (62%), and Cancer SIGVAR (57%) (Fig. 4). These results show that HerediClassify is the best performing tool on a HBOC dataset. This remains true when removing *BRCA1* and *BRCA2* variants from the dataset (Supplemental Figure 4).

vaRHC failed to classify 6 out of the 130 selected variants from HerediVar.

### Comparison HerediClassify results to consensus classification

**Fig. 5:**
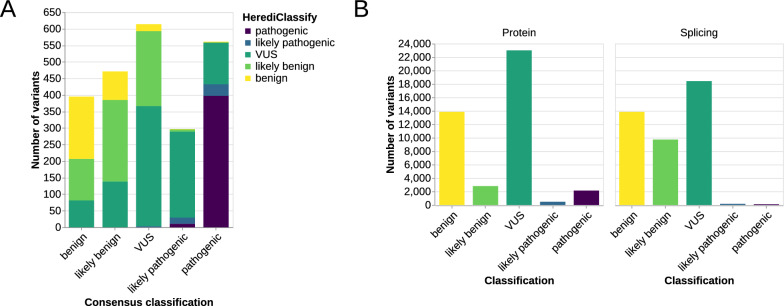
**A** Bar chart plotting the consensus classification in HerediVar by class. In color, the classification provided by HerediClassify is shown. **B** Classification results of HerediClassify on the HerediVar database. Classification based on protein evidence shown on the left and classification based on splicing evidence on the right

The VUS task force has assigned a consensus classification to 2,396 variants in HerediVar, accounting for 5.4% of variants (November 2024). For the comparison between HerediClassify and the consensus classification, the two classifications HerediClassify computes are joined into one final classification (Fig. 5A).

79% of variants with a benign consensus classification and 71% of variants with a likely benign consensus classification were classified as either likely benign or benign by HerediClassify (Supplemental Table 4). Variants with a pathogenic consensus classification were classified as either likely pathogenic or pathogenic in 77% of cases. Only 10% of variants with a likely pathogenic consensus classification were classified as either likely pathogenic or pathogenic; most (88%) were classified as VUS. This is due to lack of machine-readable data.

11 (0.5%) variants were classified as (likely) benign, but the consensus classification was (likely) pathogenic (Supplemental Table 5). Of these, 3 variants met the BA1 criterion. 8 out of 11 variants were classified as likely benign based on splice evidence and classified as VUS based on protein evidence. When summarizing the two classifications from HerediClassify for the comparison to the consensus classification, a non-VUS classification based on either protein or splicing evidence, is sufficient for a variant to have a final non-VUS classification, causing the final classification to be likely benign in these cases.

9 out of these 11 variants in *CHEK2*, *MSH2*, *RAD51D*, *PMS2*, and *BRIP1* have a discordant classification as (likely) benign due to the threshold used for BA1 (0.001%) and BS1 (0.0001%). All variants with discordant classifications by HerediClassify based on population allele frequency are located in moderate risk genes. The remaining two variants in *PALB2* were classified as benign based on a combination of BP1 and BP4.

### Results of HerediClassify on HerediVar

HerediClassify provides a non-VUS classification for 63% of variants, with 56% of variants classified as (likely) benign and 7% of variants as (likely) pathogenic (Fig. 5B). Of the 7% of variants classified as (likely) pathogenic, 6% were classified based on protein evidence and 1% were classified based on splicing evidence. Classification as (likely) benign was assigned to 39% of the variants based on protein evidence and for 56% based on splicing evidence, indicating that most variants were classified based on both protein and splice evidence.

## Discussion

The classification of genetic variants is a complex process, requiring multiple types of evidence and knowledge of gene-specific guidelines and criteria-specific recommendations. As a result, variant classification has emerged as the bottleneck in genetic diagnostics. In order to mitigate this bottleneck, tools that support experts in variant classification are urgently needed. We present HerediVar and HerediClassify, two tools for streamlining and expediting the classification of variants by experts.

HerediVar is a web application for variant annotation and classification. All relevant metadata for educated variant classification is made available in a user-friendly web interface. Uniquely, HerediVar allows clinical researchers to collaborate, discuss, and decide on a consensus classification. HerediVar also interfaces directly with HerediClassify an algorithm which provides automated classification criterion suggestions to support experts in variant classification. Both tools implement the ACMG/AMP guidelines for variant classification and gene-specific recommendations for HBOC risk genes. 19 out of the 28 ACMG/AMP criteria are automated in HerediClassify due to unavailability of machine-readable data for de-novo status, allelic data, and phenotype.

HerediClassify offers a strict separation between evidence for variant effect on protein and splicing level, as recommended by Walker et al. [39]. To our knowledge, no other tools offer such a clear separation between evidence types. HerediClassify’s separation of evidence types removes ambiguity for criteria like PS1 that can be applied for protein and splicing evidence. This improves variant classification experts’ confidence in the classification results.

HerediClassify was validated using 721 variants from the ClinGen Evidence Repository and achieved an average F1-score of 93% across all criteria. The performance of HerediClassify varies between criteria. The main causes for the discrepancies are the availability of data and updates to variant classification guidelines or databases. Due to data availability, evidence from functional assays is only implemented for *BRCA1* and *BRCA2* at the moment. By expanding the functional assays annotated in HerediVar with functional data for *TP53* [16, 21] or *RAD51C* [29], evidence from functional assays can be incorporated into variant classification for additional genes. In accordance with current recommendation, HerediClassify only uses one tool for splicing prediction and one tool for protein effect prediction, while some gene-specific recommendations call for use of multiple prediction tools [32].

For the validation, we performed reannotation of the dataset with gnomAD v2.1.1, in accordance with the gnomAD version used in the ClinGen Evidence Repository, whilst HerediVar uses v3.1.2 by default. HerediVar uses gnomAD v3.1.2 by default because of the better representation of intronic variants in gnomAD v3.1.2. In the gene-specific recommendation guidelines, only the ATM guidelines specify a gnomAD version (v2.1.1), although a future update to v4 has already been discussed in the paper [13].

In our benchmarking study using 130 variants from HBOC risk genes, we were able to show that HerediClassify outperforms all other tested variant classification tools, including vaRHC and Cancer SIGVAR. Whilst HerediClassify, outperforms vaRHC and Cancer SIGVAR even when excluding *BRCA1* and *BRCA2*, the fact that these tools do not implement gene-specific recommendations for *BRCA1* and *BRCA2* decreases their performance. *BRCA1* and *BRCA2* are the most commonly affected genes in HBOC making up more than 30% of variants in the benchmarking dataset [5]. This highlights the importance and effectiveness of utilizing gene-specific variant classification guidelines, as previously shown for manual variant classification [31, 43]. Furthermore, differences in the version of the database like gnomAD and ClinVar, as well as the use of different prediction tools, can cause differences between the results of the classification tools. Additionally, different underlying assumptions can cause differences in the application of criteria; TAPES, for instance, applies BS3 and PS3 based on pathogenic classification on ClinVar for variants with evidence-level “practice guidelines” or “reviewed by expert panel” [42].

The main limitation for all automated variant classification is the availability of machine-readable data, including data from functional assays, segregation data, de novo status, allelic information, and multifactorial likelihoods. While access to functional data has improved through Multiplexed Assays of Variant Effect (MAVE) or Multiplexed Parallel Reporter Assays (MPRA), accessing data from smaller, non-high-throughput studies remains a significant challenge [35]. Literature mining tools, such as VarChat [10] or LitVar 2.0 [2], can assist in screening the literature for additional evidence, including small functional studies and segregation studies, although literature still needs to be assessed manually. Annotation of literature sources is integrated into HerediVar through LitVar 2.0 (Supplemental Table 1). To address this limitation, large databases consolidating evidence from across the literature would be required, especially for multifactorial likelihood ratios and cosegregation studies.

Variant classification guidelines are constantly evolving with updates to existing guidelines being published, for instance for *TP53*, where version 2 was just published [38]. Additionally, the ACMG/AMP recommendations version 4 will replace existing criteria with decision trees [8]. To accommodate these updates, automated classification algorithms have to be regularly extended and updated. HerediClassify’s modular design allows for such adaptation to be made easily.

The modularity of HerediVar’s annotation services and HerediClassify’s input enables easy integration of novel annotation tools. For instance, gnomAD version 4 [17] and novel pathogenicity prediction tools like AlphaMissense have become available [7]. Further annotations tailored to intronic regions like promotor or enhancer regions will become necessary as genome sequencing becomes more common [18].

HerediVar and HerediClassify are designed for short germline variants. Although HerediVar allows inserting copy number variants, their support is rather rudimentary and will be extended. For example, more annotations like promoter or enhancer regions are required. Due to the modular design of HerediClassify, recommendations for the classification of copy number variants [34] and somatic variant classification [19] can be easily integrated.

Because of updates to guidelines and the availability of new evidence like functional assays, variants should be reclassified two years after initial classification [40]. This exacerbates variant classification as the bottleneck in genetic diagnostics. Automated variant classification tools like HerediClassify can support experts in variant classification, through the preselection of applicable criteria, and reclassification, by highlighting variants that have a point score close to a classification cut-off, that are more likely to be classified upon manual inspection of the variant. In HerediVar these variants can be easily identified through filtering.

We present HerediVar a database designed to support variant classification and HerediClassify an algorithm for automated variant classification. With over 50 active users across 26 genomic research centers in Germany and nearly 40,000 variants–including 2,369 with consensus classifications–HerediVar is a powerful solution for variant classification. All variants with a consensus classification including applied criteria are available for download on the HerediVar website. The integration of HerediVar and HerediClassify streamlines the classification of variants and provides crucial support to classification experts at the VUS task force of the GC-HBOC. At the time of publication our tools are already in productive use by the VUS task force for over two years. Throughout this time many adjustments have been made to the web application which is enabled by the constant feedback loop from our users. Because of this, HerediVar and HerediClassify are under constant adjustment to the newest changes in the field. While initially developed for HBOC, this use-case serves as an example for the broader potential of these tools. Due to their modularity, HerediVar and HerediClassify can be easily expanded to support variant classification in genetic diagnostics as a whole.

## Additional file

Supplementary file 1 (xlsx 64 KB)

Supplementary file 2 (pdf 333 KB)

## Data Availability

All variants with consensus classification are available for download on HerediVar website (https://heredivar.uni-koeln.de/downloads). Additionally, consensus classification are uploaded to ClinVar in regular intervals (see https://www.ncbi.nlm.nih.gov/clinvar/submitters/506864). The source code is available on GitHub for HerediVar (https://github.com/GC-HBOC/HerediVar) and HerediClassify (https://github.com/akatzke/HerediClassify).
