# Supplementary material for: HerediVar and HerediClassify: tools for streamlining genetic variant classification in hereditary breast and ovarian cancer
Source: Hum Genomics. 2025 Jul 4;19:76. doi: 10.1186/s40246-025-00787-w (PMC12228362; doi:10.1186/s40246-025-00787-w)
Supplement: Supplementary file 2 — (pdf 333 KB) [file 40246_2025_787_MOESM2_ESM.pdf]

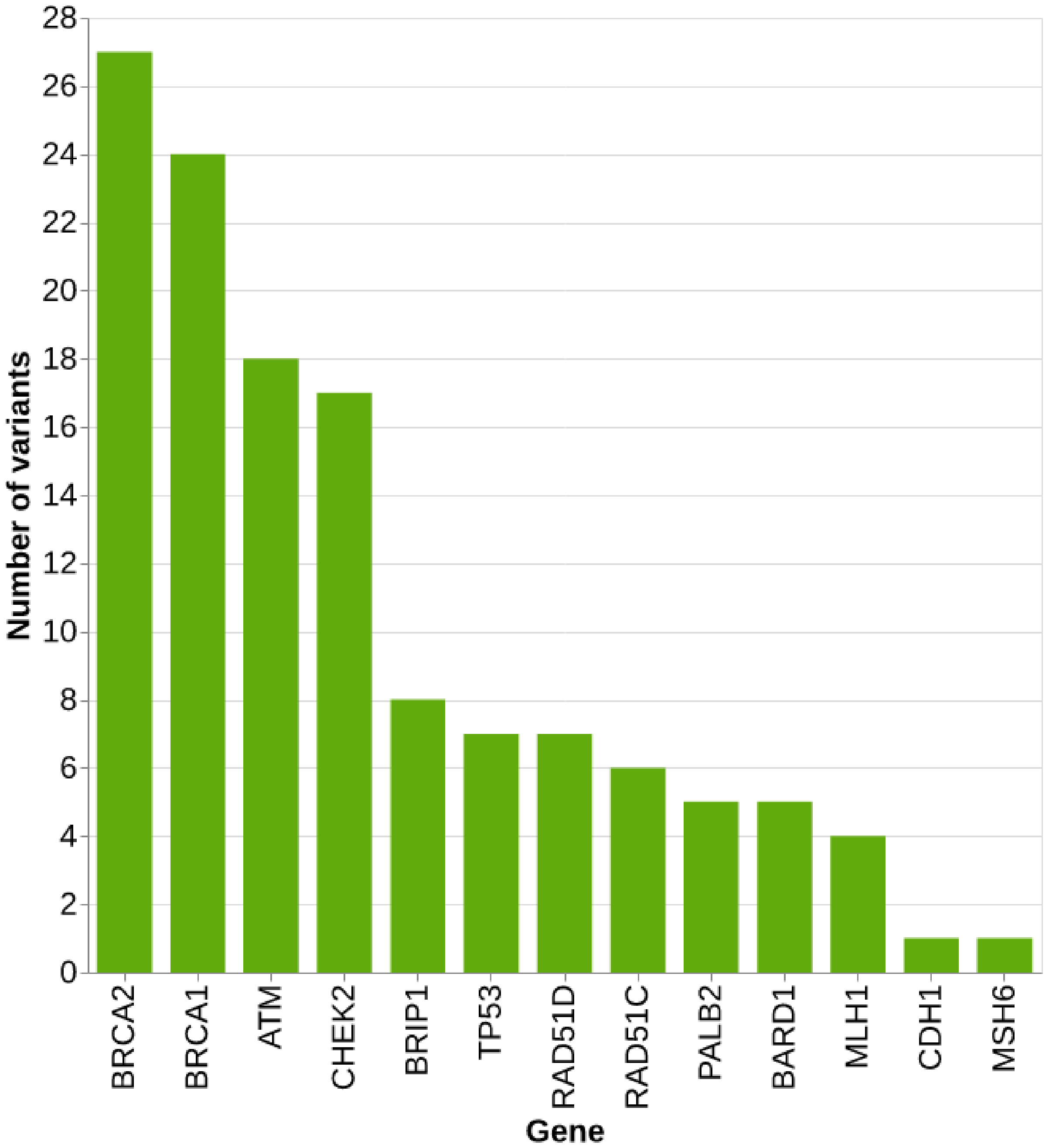

Supplemental Figure 1: Distribution of genes in the 130 variants selected from the HerediVar database that were classified by the VUS Taskforce, the variant classification expert panel of the German Consortium of Hereditary Breast and Ovarian Cancer. This dataset was used for the tool comparison between HerediClassify, vaRHC, TAPES, InterVar, VarSome and Cancer SIGVAR.

Create a consensus classification

It appears that you already have a classification for this variant and scheme. You can edit it here.

**Variant**	chr17-43106477-C-T
**HGVS**	BRCA1 ENST00000357654 c.191G>A p.Cys64Tyr
**Links**	HerediVar UCSC gnomAD v3 gnomAD v4 VarSome dbSNP ClinVar

Select type of classification

Consensus classification

Classification scheme

ClinGen ENIGMA BRCA1 v1.1.0

Go here for more details about the currently selected scheme.

Preselect criteria

Pathogenic evidence

PVS1	PS1	PM1	PP1
	PS2	3 PM2_sup	PP2
	3 PS3	PM3	2 PP3
	PS4	PM4	2 PP4_vstr
		PM5	PP5
		PM6	

Benign evidence

BP1_str	BS1	BA1
BP2	BS2	
BP3	BS3	
BP4	BS4	
BP5		
BP6		
BP7		

Classification based on selected criteria:

4

selected PP3

Description:

Original ACMG Summary
Multiple lines of computational evidence support a deleterious effect on the gene or gene product (conservation, evolutionary, splicing impact, etc.).
Caveat: As many in silico algorithms use the same or very similar input for their predictions, each algorithm should not be counted as an independent criterion. PP3 can be used only once in any evaluation of a variant.

Gene-Specific modifications:
Apply PP3 for missense or in-frame insertion, deletion or delins variants inside a (potentially) clinically important functional domain and predicted impact via protein change (BayesDel no-AF score >=0.28). As justified in the appendices, (potentially) clinically important functional domains are defined as: BRCA1 RING aa 2-101; BRCA1 coiled-coil aa 1391-1424; BRCA1 BRCT repeats aa 1650-1857.
Apply PP3 for predicted splicing (SpliceAI >=0.2) for silent, missense/in-frame (irrespective of location in clinically important functional domain) and for intronic variants outside of donor and acceptor 1,2 sites.

Instructions:
See Specifications Figure 1A for process to apply codes according to variant type, location and predicted bioinformatic impact.

User selections:

User	Affiliation	Strength	Evidence	Selected	Date	Copy
search...	search...	search...	search...	search..	search...	
Arno Nym	Anonymous	supporting	BayesDel no AF: 0,557	selected	2024-12-15 15:45:22	
Jon Doe	lab1	supporting	This BRCA1 missense variant is within a key functional domain and the computational predictor BayesDel (noAF) gives a score of 0.557, above the recommended threshold of 0.28 for prediction of impact on BRCA1 function via protein change. SpliceAI predictor score of 0.00 suggests that the variant has no impact on splicing (score threshold <0.10)	selected	2024-12-08 14:11:12	
Max Mustermann	lab2	supporting	REVEL (v2021-05-03): Score: 0.954.	selected	2024-12-15 15:38:05	

Evidence:

This BRCA1 missense variant is within a key functional domain and the computational predictor BayesDel (noAF) gives a score of 0.557, above the recommended threshold of 0.28 for prediction of impact on BRCA1 function via protein change. SpliceAI predictor score of 0.00 suggests that the variant has no impact on splicing (score threshold

Final classification

5

Comment (optional)

comment

Literature (optional)

Add blank row | Add from text mining | Add from user

PMID	Text passage	Remove
	Nothing to show	

Submit classification

Supplementary Figure 2: The consensus classification page for rs5585183. PP3, PS3 and PM2_sup are selected. Numbers in the upper left corner of each criterion indicate the number of users who selected the respective criterion for this variant. Faded criterion buttons cannot be selected (either because the scheme prevents it or selected criterions are mutually exclusive, like PVS1 and PP3). The right side shows a preview of the variant classification under the currently selected criteria. The "preselect criteria" button in the upper right corner allows copying the HerediClassify classification directly in this user interface for m annual curation.

PP3 has been selected, thus, the current view shoes the instruction on how to select the PP3 criterion. Below that all individual user selections of the PP3 criterion are shown. The final classification, a general comment along with literature evidence can be provided in the last form input fields.

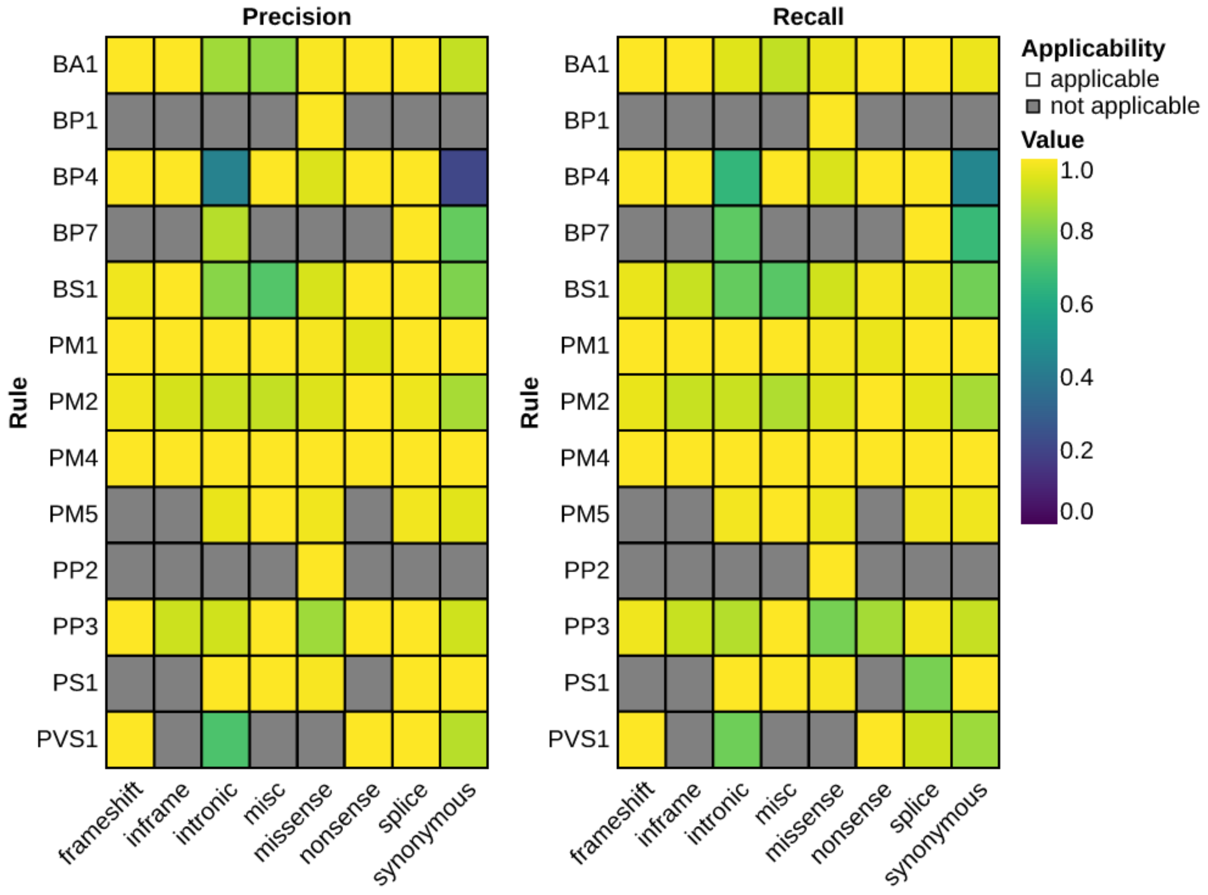

Supplemental Figure 3: Heatmap showing precision (left) and recall (right) of HerediClassify on the validation dataset from the ClinGen Evidence Repository. The performance is depicted for every rule and for every variant type; frameshift (n = 93), inframe (n=12) [including inframe deletions and insertions], intronic (n=62) [intronic variants that are not splice acceptor or splice donor variants], misc (n=42), missense (n=345), nonsense (n=67), splice (n=54) [defined as splice acceptor and splice donor variants], and synonymous (n=46). BP3 is not applicable according to all gene-specific guidelines. Results for PP1 and BS4 are excluded due to unavailability of data. BS3, PS3, and BS2 are not shown, as they are only implemented for a small subset of genes, and results would therefore be misleading. Criteria that are not applicable to a variant type are shown in grey.

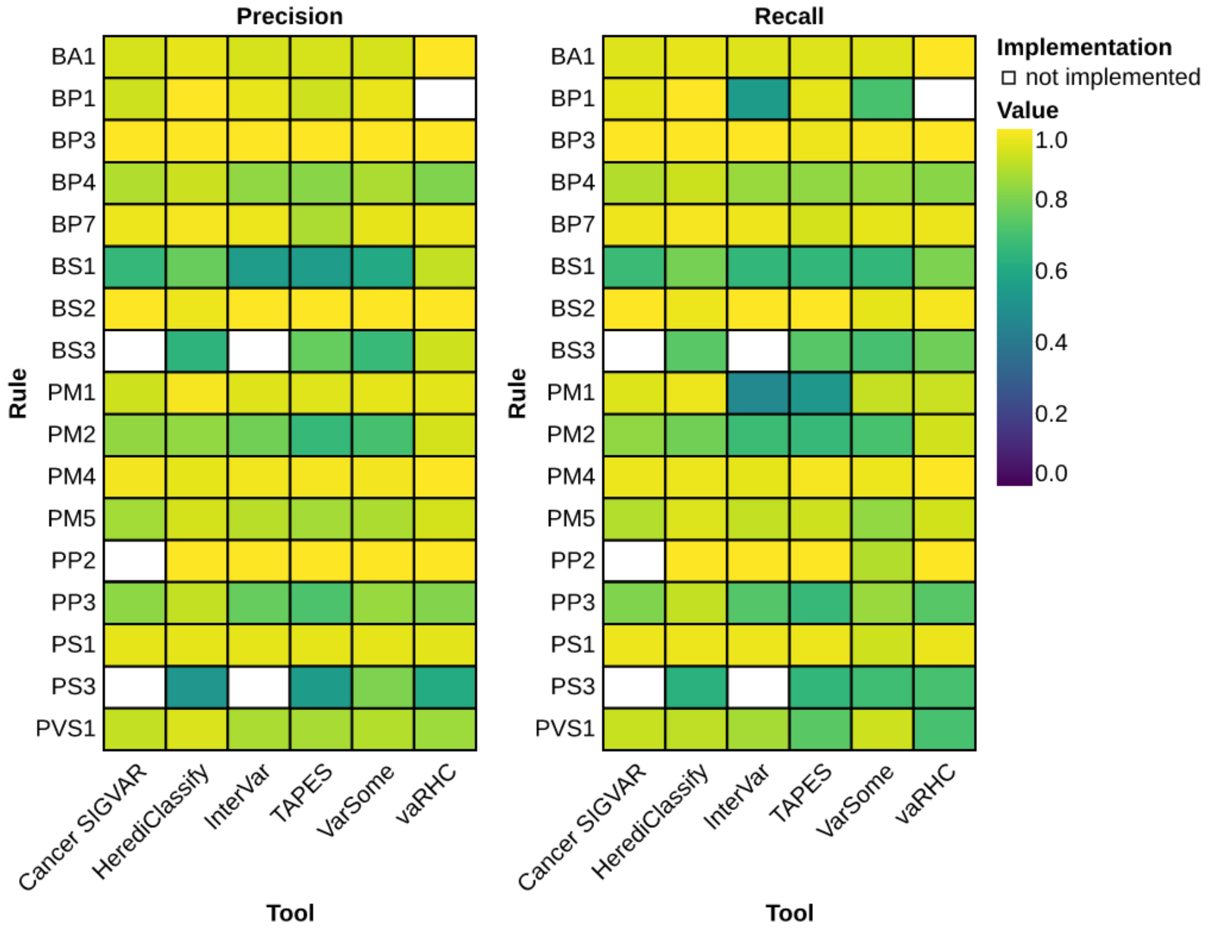

Supplemental Figure 4: Heatmap showing precision (left) and recall (right) for HerediClassify, vaRHC, VarSome, Cancer SIGVAR, TAPES and InterVar for every rule. Variants located in BRCA1 and BRCA2 were excluded for the purpose of this analysis. Performance metrics were calculated taking the evidence strength into account. White fields indicate rules that have not been implemented by the respective tool.
